# Analysis of correlative risk factors for radiation-induced hypothyroidism in head and neck tumors

**DOI:** 10.1186/s12885-023-11749-7

**Published:** 2024-01-02

**Authors:** Chan Wang, Yanjie Hou, Lili Wang, Ye Yang, Xianfeng Li

**Affiliations:** Department of Radiation Oncology, The First Hospital of Shanxi Medical University, Taiyuan, China

**Keywords:** Head and neck cancer, Radiotherapy, Injury of thyroid, Radiation-induced hypothyroidism

## Abstract

**Objective:**

The aim of the study is to identify clinical and dosimetric factors that could predict the risk of radiation-induced hypothyroidism(RIHT) in head and neck cancer(HNC) patients following intensity-modulated radiotherapy(IMRT).

**Methods:**

A total of 103 HNC patients were included in our study. General clinical characteristic and dosimetric data of all recruited patients were analyzed, respectively. The univariate and multivariate logistic regression anlalysis were successively conducted to identify optimal predictors, which aim to construct the nomogram. And the joint prediction was performed.

**Results:**

The incidence of patients with HNC was 36.9% (38/103). Among the clinical factors, gender, N stage, chemotherapy, frequency of chemotherapy and surgery involving the thyroid were related to RIHT. Logistic regression analysis showed that thyroid volume, Dmean, VS_45_, VS_50_, VS_60_ and V_30,60_ were independent predictors of RIHT, which were also incorporated in the nomogram. An AUC of 0.937 (95%CI, 0.888–0.958) also was showed outstanding resolving ability of the nomogram. When the volume of the thyroid was greater than 10.6 cm^3^, the incidence of RIHT was 14.8%, and when the volume of the thyroid was equal to or smaller than 10.6 cm^3^, the incidence was 72.5%. The incidence rates of RIHT in the group with VS_60_≦8.4cm^3^ and VS_60_ > 8.4cm^3^ were 61.4% and 19.3%, respectively.

**Conclusions:**

Thyroid volume and thyroid VS_60_ are independent predictors of RIHT in patients with HNC. Moreover, more attention should be paid to patients with thyroid volume ≤ 10.6cm^3^. Thyroid VS_60_ > 8.4cm^3^ may be a useful threshold for predicting the development of RIHT. The nomogram conducted by the research may become a potential and valuable tool that could individually predict the risk of RIHT for HNC patients.

## Introduction

Head and neck cancers account for 6% of all global malignant tumors [1], and radiotherapy is a major treatment for HNC [2]. Patients with HNC are often diagnosed at an advanced stage; thus, most of the neck is included in the radiation field during radiation therapy. Because the thyroid is located in the middle of the neck, it will inevitably be exposed to radiation, which can lead to insufficiency and biochemical changes. Radiation-induced thyroid injury is a common side effect after radiotherapy in the neck region, including hypothyroidism, hyperthyroidism, benign thyroid nodules, and thyroid cancer. Among them, RIHT is the most common [3]. Hypothyroidism increases the risk of atherosclerosis [4], thereby increasing the incidence and mortality of cardiovascular and cerebrovascular diseases and ultimately greatly reducing the quality of life of patients [5–7]. According to relevant literature reports, the incidence of hypothyroidism 2 years after radiotherapy is 40% to 50%, of which subclinical hypothyroidism accounts for 70% and clinical hypothyroidism accounts for 30% [8]. The 2010 quantitative analysis report of clinical normal tissue effects did not mention the limiting dose of the thyroid gland [9]; thus, many studies are currently devoted to exploring the risk factors for hypothyroidism after radiotherapy for HNC and establishing normal tissue complication probability to ensure a limited dose of the thyroid. However, relevant studies have not yet reached consensus.

## Materials and methods

### Patient evaluation

Overall, 103 patients with HNC were recruited, including nasal cavity and sinus cancer, nasopharyngeal cancer, oral cancer, oropharyngeal cancer, hypopharyngeal cancer, laryngeal cancer, salivary gland cancer and cervical esophagus cancer. According to the patient's past medical history and the determination of thyroid function before radiotherapy, patients with hypothalamic disease, pituitary disease, abnormal thyroid function, severe heart disease, severe liver and kidney dysfunction, and a history of radiotherapy or chemotherapy were excluded.

### Radiation treatment

Patients were immobilized with a thermoplastic head-and-neck mask that included the shoulders to ensure reproducibility of radiotherapy. Computed tomographic simulation was performed in all patients, and the patients were planned using the Eclipse treatment-planning system with 6-MV photons. All patients received image-guided IMRT(Intensity modulation radiated therapy). Position verification was performed at least once a week by EPID (Electronic Portal Imaging Device), and the verification error was less than 3 mm.

The thyroid gland was contoured manually on CT (Computed Tomography) images, and the absolute volume of the thyroid; mean dose; maximum dose; minimum dose of the thyroid; and the percent of thyroid volume receiving more than 30, 35, 40, 45, 50, 55, and 60 Gy (V_30_, V_35_, V_40_, V_45_, V_50_, V_55_, and V_60_) were then collected from dose-volume histograms (DVHs). According to V_x_, we counted the absolute volume of thyroid sparing at 30, 35, 40, 45, 50, 55, and 60 Gy (VS_30_, VS_35_, VS_40_, VS_45_, VS_50_, VS_55_, and VS_60_) and obtained the percentage of thyroid volume receiving a-b Gy (V_a,b_).

### Chemotherapy treatment

Concurrent chemotherapy patients received nab-paclitaxel and/or cisplatin/ lobaplatin chemotherapy. The regimen is nab-paclitaxel + cisplatin, single-agent cisplatin, and single-agent lobaplatin.

### Follow-up

Before and after radiotherapy, all patients underwent thyroid function tests, including serum thyroid-stimulating hormone (TSH), free triiodothyronine (FT3), and free thyroxine (FT4). Hypothyroidism includes clinical hypothyroidism and subclinical hypothyroidism. Clinical hypothyroidism was defined as TSH above the normal range (0.380–4.340) and an FT4 concentration below the normal range (0.81–1.89). Subclinical hypothyroidism was defined as TSH above the normal range and FT4 concentrations within the normal range. The time to onset of hypothyroidism was defined as the interval between the end of radiotherapy and the first recorded abnormal TSH laboratory value.

### Statistical methods

Statistical description of measurement data used the means ± standard deviation. SPSS software was used to perform t tests or Wilcoxon rank-sum tests for measurement data and chi-square tests or Fisher's exact tests for count data. Only characteristics significantly different in univariate analysis were included in the logistic regression model for constructing the nomogram. Receiver operating characteristic (ROC) curves were used to determine the limiting value of risk factors for the development of RIHT. MedCalc software was used to assess the cumulative incidence of hypothyroidism and compare survival curves. *P* ≤ 0.05 was considered to be statistically significant.

## Results

### Characteristics

Among the 103 patients enrolled, 38 developed hypothyroidism, of whom subclinical hypothyroidism occurred in 18 patients and clinical hypothyroidism in 20 patients. The average age was 58 years, and the median follow-up time was 10 months. The shortest follow-up time was 3 months, and the longest was 30 months. There were 38 cases in the hypothyroid group and 63 cases in the euthyroid group. The incidence of hypothyroidism was 36.9% (38/103), of which subclinical hypothyroidism accounted for 47.4% (18/38) and clinical hypothyroidism for 52.6% (20/38). The results of this study show that the earliest occurrence of RIHT in HNC was 3 months after radiotherapy, the latest was 27 months after radiotherapy, and the average occurrence time was 7.3 months after radiotherapy.

Of 103 patients with HNC, 21 were female, and 80 were male. The incidence of RIHT in female patients was 61.9% (13/21), and the incidence in male patients was 31.2% (25/80). The risk of hypothyroidism in women was significantly higher than that in men, and there were significant difference between gender and RIHT (*P* = 0.01).

Gender, N stage, chemotherapy, frequency of chemotherapy, and operation involving the thyroid gland were significantly associated with the occurrence of RIHT in patients with head and neck tumors (Table 1). Three factors, including N stage, chemotherapy, and surgery involving the thyroid, all affected the size of the radiation field of the patients.

**Table 1:** Statistical analysis of the general clinical characteristics of 101 patients who received head and neck tumor radiotherapy

Variable	Euthyroid (*n* = 63)	Hypothyroid ( *n* = 38)	*P*
Age (years)	58.6 ± 12.5	58.6 ± 14.3	0.883
Gender			0.01
Male	55	25	
Female	8	13	
T stage			0.938
T1-T2	24	24	
T3-T4	39	22	
N stage			0.05
N0-N1	44	18	
N2-N3	19	18	
Overall stage			0.457
I-II	16	7	
III-IV	47	30	
Chemotherapy			0.007
no	32	9	
yes	31	29	
Frequency of chemotherapy	1.7 ± 2.1	2.5 ± 2.1	0.021
Tumor site			0.058
Nasal cavity and sinus	10	4	
Nasopharynx	2	8	
Oral cavity	13	7	
Oropharynx	4	2	
Larynx	21	9	
Hypopharynx	7	5	
Salivary gland	6	1	
cervical esophagus cancer	0	2	
Targeted therapy			0.591
no	45	29	
yes	18	9	
Surgery			0.189
no	20	17	
yes	43	21	
Surgery involving thyroid			0.027
no	62	33	
yes	1	5	

### Dose-volume parameters

The mean thyroid volume in the hypothyroid group was 9.53 ± 4.26 Gy, and the mean volume in the euthyroid group was 17 ± 9.0 Gy. Numerous dosimetric parameters were significantly different between the euthyroid and hypothyroid groups, such as thyroid volume, Dmean, Dmin, thyroid V_50_, VS_30_, VS_35_, VS_40_, VS_45_, VS_50_, VS_55_, VS_60_, V_35,60_ and V_30,60_ (Table 2). Others were not found to significantly affect the development of hypothyroidism.

**Table 2:** Analysis of RIHT-related radioactive parameters in 101 patients with head and neck tumors

Variable	Euthyroid (*n* = 63)	Hypothyroid (*n* = 38)	*P*
Thyroid volume	17 ± 9.0	9.53 ± 4.26	0.000
Dmean	41.7 ± 19.3	53.2 ± 7.9	0.013
Dmin	23.8 ± 19.0	36.9 ± 12.2	0.001
Dmax	59.7 ± 16.8	65.1 ± 4.0	0.330
V_30_	69.9 ± 37.2	94.3 ± 16.8	0.170
V_35_	68.0 ± 37.7	93.4 ± 17.4	0.446
V_40_	65.1 ± 37.8	91.2 ± 17.8	0.084
V_45_	60.1 ± 36.9	85.0 ± 20.9	0.365
V_50_	53.3 ± 36.0	74.0 ± 23.2	0.027
V_55_	41.0 ± 34.6	52.7 ± 30.7	0.219
V_60_	24.8 ± 28.0	29.0 ± 27.5	0.458
VS_30_	5.0 ± 7.8	0.6 ± 1.7	0.000
VS_35_	5.3 ± 8.2	0.7 ± 1.8	0.000
VS_40_	5.8 ± 8.6	0.9 ± 1.9	0.001
VS_45_	6.7 ± 8.9	1.5 ± 2.1	0.000
VS_50_	7.1 ± 7.4	2.7 ± 2.5	0.001
VS_55_	8.8 ± 7.3	4.9 ± 4.0	0.002
VS_60_	12 ± 8.5	7.1 ± 4.4	0.000
V_30,35_	1.9 ± 3.2	0.9 ± 1.7	0.225
V_35,40_	2.9 ± 4.2	2.3 ± 3.4	0.736
V_40,45_	5.0 ± 5.5	6.2 ± 10.6	0.460
V_45,50_	6.8 ± 6.6	11.1 ± 9.3	0.279
V_50,55_	12.4 ± 13.2	21.3 ± 19.5	0.099
V_55,60_	16.1 ± 13.9	23.7 ± 19.3	0.551
V_30,40_	4.8 ± 6.5	3.2 ± 4.5	0.955
V_35,45_	7.9 ± 8.8	8.5 ± 12.9	0.535
V_40,50_	11.8 ± 11.3	17.2 ± 17.1	0.085
V_45,55_	19.2 ± 17.9	32.3 ± 27.0	0.062
V_50,60_	28.5 ± 21.9	45.0 ± 25.0	0.403
V_30,45_	9.8 ± 10.6	9.3 ± 13.5	0.994
V_35,50_	14.8 ± 14.4	19.5 ± 19.1	0.213
V_40,55_	24.2 ± 21.5	38.5 ± 30.1	0.105
V_45,60_	35.3 ± 25.8	56.0 ± 29.4	0.226
V_30,50_	16.6 ± 15.9	20.4 ± 19.5	0.119
V_35,55_	27.1 ± 23.8	40.8 ± 31.2	0.158
V_40,60_	40.3 ± 29.2	62.2 ± 30.7	0.107
V_30,55_	29.0 ± 24.9	41.6 ± 31.4	0.195
V_35,60_	43.3 ± 30.6	64.5 ± 31.4	0.043
V_30,60_	45.1 ± 30.9	65.3 ± 31.3	0.026

Abbreviations: *Dmean* Mean dose, *Dmin* Minimum dose, *Dmax* Maximum dose, *V*_*30*_* V*_*35*_* V*_*40*_*, V*_*45*_*, V*_*50*_*, V*_*55*_*, V*_*60*_ the percent of thyroid volume receiving more than 30, 35, 40, 45, 50, 55, 60 Gy, *VS*_*30*_*, VS*_*35*_*, VS*_*40*_*, VS*_*45*_*, VS*_*50*_*, VS*_*55*_*, VS*_*60*_ the absolute volumes of thyroid spared from more than 30, 35, 40, 45, 50, 55, 60 Gy, *V*_*30,35*_*, V*_*35,40*_*, V*_*40,45*_*, V*_*45,50*_*, V*_*50,55*_*, V*_*55,60*_*, V*_*30,40*_*, V*_*35,45*_*, V*_*40,50*_*, V*_*45,55*_*, V*_*50,60*_*, V*_*30,45*_*, V*_*35,50*_*, V*_*40,55*_*, V*_*45,60*_*, V*_*30,50*_*, V*_*35,55*_*, V*_*40,60*_*, V*_*30,50*_*, V*_*35,55*_*, V*_*40,60*_*, V*_*30,55*_*, V*_*35,60*_*, V*_*30,60*_ percentage of thyroid volume receiving 30–35, 35–40, 40–45, 45–50, 50–55, 55–60,30–40, 35–45, 40–50, 45–55, 50–60, 30–45, 35–50, 40–55, 45–60, 30–50, 35–55, 40–60, 30–50, 35–55, 40–60, 30–55, 40–60, 30–55, 35–60, 30–60 Gy. *Statistically significant at the level of 5%

**Fig. 1:**
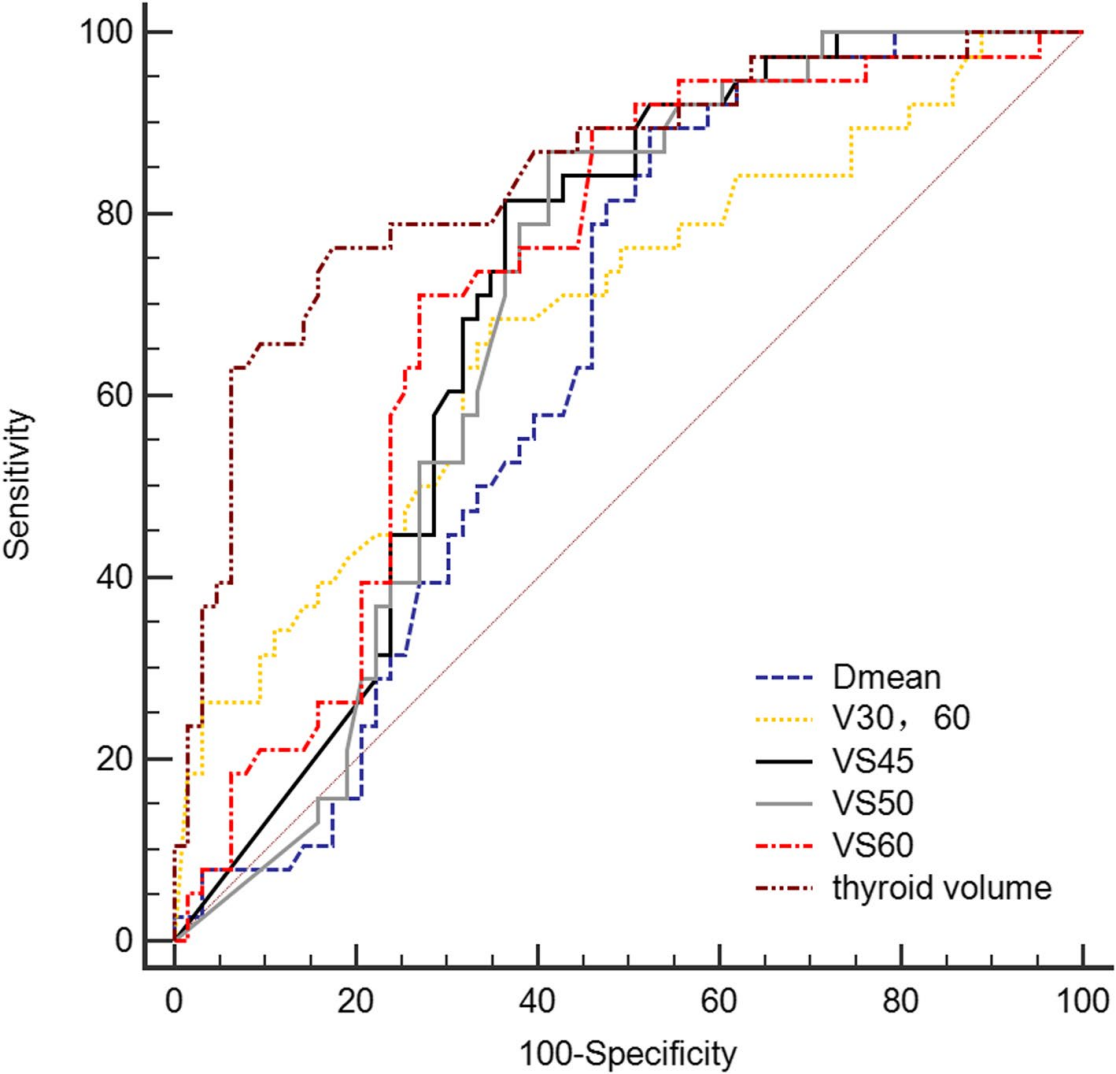
Comparison of ROC with Dmean, thyroid volume, VS_45_, VS_50_, VS_60_ and V_30,60_ Abbreviations: Dmean: mean dose; V30,60: percentage of thyroid volume receiving 30–60 Gy; VS45, VS50, VS60: the absolute volumes of thyroid spared from more than 45, 50, 60 Gy

The logistic regression analysis was performed to further test the association of the factors with RIHT (Table 3). In clinical factors, use of chemotherapy and thyroid volume remained the most important factors in predicting RIHT. The results of our study further confirmed a clear dose-dependent relationship for RIHT: Dmean (*P* = 0.001), VS_45_ (*P* = 0.016), VS_50_ (*P* = 0.032), VS_60_ (*P* = 0.018) and V_30,60_ (*P* = 0.009) were independent predictors of RIHT. With regard to ROC analysis of the dosimetric parameters, the area under the curve (AUC) was significantly different from 0.05 for the above parameters, and the difference was greater for thyroid volume (AUC = 0.841, *P* < 0.001) and VS_60_ (AUC = 0.724, *P* = 0.011) (Fig. 1). The thyroid volume and thyroid VS_60_ cutoffs were 10.6 cm^3^ and 8.4cm^3^, respectively. Thyroid radiosensitivity decreased with increasing thyroid volume, and thyroid volume ≤ 10.6 cm^3^ was an independent predictor of RIHT. When the volume of the thyroid was greater than 10.6 cm^3^, the incidence of RIHT was 14.8%, and when the volume of the thyroid was equal to or smaller than 10.6 cm^3^, the incidence of RIHT was 72.5%. According to thyroid volume, the incidence of different subgroups was significantly different (*P* = 0.000) (Table 4). A threshold of 8.4cm^3^ for VS_60_ was identified to classify patients into high-risk and low-risk groups for the development of RIHT. The incidences of RIHT in the group with VS_60_≦8.4cm^3^ and VS_60_ > 8.4cm^3^ were 61.4% and 19.3%, respectively. There were significant differences between the two subgroups of thyroid VS_60_ (*P* = 0.000) (Table 4).

**Table 3:** Logistic regression analysis of factors influencing hypothyroidism in patients with head and neck tumors

Variable	B	SE	*P*	OR(95%CI)
Thyroid volume	-1.312	0.377	0.000	0.269 (0.129–0.563)
chemotherapy	2.140	1.026	0.037	8.500(1.138–63.506)
VS_45_	-1.762	0.728	0.016	0.172 (0.041–0.715)
VS_50_	1.574	0.735	0.032	4.827 (1.142–20.400)
VS_60_	0.826	0.348	0.018	2.284 (1.155–4.519)
Dmean	0.245	0.075	0.001	1.278 (1.103–1.481)
V_30,60_	-0.107	0.041	0.009	0.898 (0.829–0.974)

*Abbreviations*: *VS*_*45*_ the absolute volumes of thyroid spared from more than 45 Gy, *VS*_*50*_ the absolute volumes of thyroid spared from more than 50 Gy, *VS*_*60*_ the absolute volumes of thyroid spared from more than 60 Gy, *Dmean* mean dose, *V*_*30,60*_ percentage of thyroid volume receiving 30–60 Gy. *Statistically significant at the level of 5%

**Table 4:** Analysis of different subgroups of 101 patients with head and neck tumors

Variable	Euthyroid (*n* = 63)	Hypothyroid (*n* = 38)	*P*
Group			0.000
Volume ≤ 10.6 cm^3^	11(27.5%)	29(72.5%)	
Volume > 10.6 cm^3^	52(85.2%)	9(14.8%)	
Group			0.000
VS_60_ ≦8.4cm^3^	17(38.6%)	27(61.4%)	
VS_60_ > 8.4cm^3^	46(80.7%)	11(19.3%)	

*Abbreviations*: *VS*_*60*_ the absolute volumes of thyroid spared from more than 60 Gy. *Statistically significant at the level of 5%

**Fig. 2:**
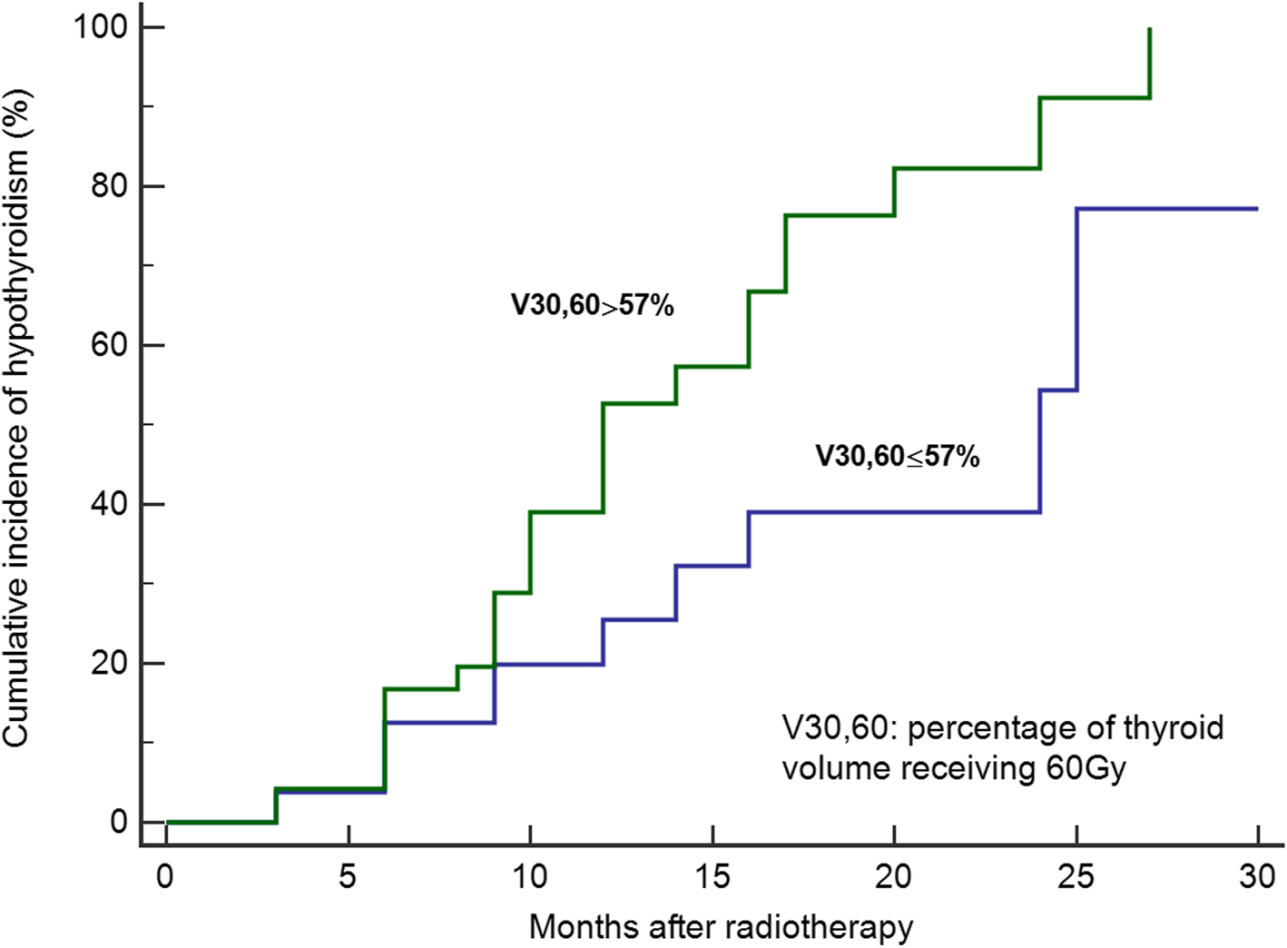
Kaplan‒Meier survival curve of different subgroups with V_30,60_

Likewise, V_30,60_ was also significant factors in logistic regression analysis. The Area under curve for V_30,60_ with a cut-off value of 57% is shown in Fig. 1. The Kaplan–Meier curve for V_30,60_ showed that patients with V_30,60_≦57% had a 2-year hypothyroidism rate of 54.3% as opposed to 91.1% for those with V_30,60_ > 57% (Fig. 2).

### Joint prediction for RIHT

Based on the above results, the joint prediction model was based on two variables: thyroid volume and thyroid VS_60_, thyroid volume and V_30,60_, thyroid VS_60_ and V_30,60_. For each component model, thyroid volume and thyroid VS_60_ (*P* = 0.000), thyroid volume and V_30,60_ (*P* = 0.000), and thyroid VS_60_ and V_30,60_ (*P* = 0.000) were significantly different between patients with and without RIHT (Table 5). The incidence of RIHT increased as the thyroid volume and VS_60_ decreased (*P* = 0.000), ranging from 17% to 86.7%. An increased trend of RIHT was observed with decreased thyroid volume while thyroid V_30,60_ increased (*P* = 0.000), and ranged from 3 to 90%. Similar trends were also found in patients with decreased thyroid VS_60_ while thyroid V_30,60_ increased (*P* = 0.000), ranging from 4 to 100%.

**Table 5:** Joint prediction for hypothyroidism

Combined factors	Euthyroid	Hypothyroid	*P*
**Combined thyroid volume and VS60**			
Volume ≤ 10.6 cm^3^ and VS_60_ ≦8.4cm^3^	4(13.3%)	26(86.7%)	0.000
Volume ≤ 10.6 cm^3^ and VS_60_ > 8.4cm^3^	7(70%)	3(30%)	
Volume > 10.6 cm^3^ and VS_60_ ≦8.4cm^3^	13(92.9%)	1(7.1%)	
Volume > 10.6 cm^3^ and VS_60_ > 8.4cm^3^	39(83%)	8(17%)	
**Combined thyroid volume and V(30,60)**			0.000
Volume ≤ 10.6 cm^3^ and V_30,60_ ≦57%	9(45%)	11(55%)	
Volume ≤ 10.6 cm^3^ and V_30,60_ > 57%	2(10%)	18(90%)	
Volume > 10.6 cm^3^ and V_30,60_ ≦57%	32(97%)	1(3%)	
Volume > 10.6 cm^3^ and V_30,60_ > 57%	20(71.4%)	8(28.6%)	
**Combined VS60 and V(30,60)**			0.000
VS_60_≦8.4cm^3^ and V_30,60_ ≦57%	17(60.7%)	11(39.3%)	
VS_60_≦8.4cm^3^ and V_30,60_ > 57%	0(0%)	16(100%)	
VS_60_ > 8.4cm^3^ and V_30,60_ ≦57%	24(96%)	1(4%)	
VS_60_ > 8.4cm^3^ and V_30,60_ > 57%	22(68.8%)	10(31.2%)	

*Abbreviations*: *VS*_*60*_ the absolute volumes of thyroid spared from more than 60 Gy, *V*_*30,60*_ percentage of thyroid volume receiving 30–60 Gy. *Statistically significant at the level of 5%

### Prognostic nomogram for predicting RIHT

**Fig. 3:**
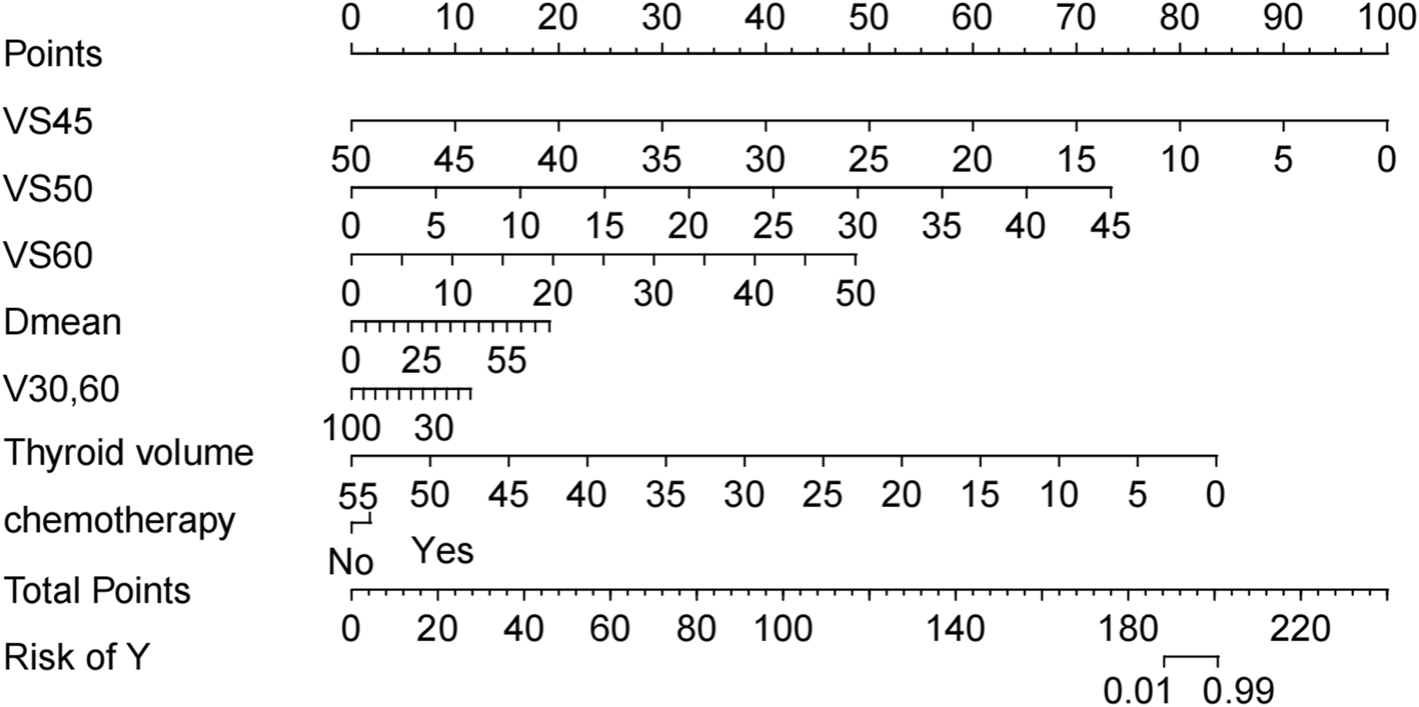
Nomogram for the probability of RIHT

**Fig. 4:**
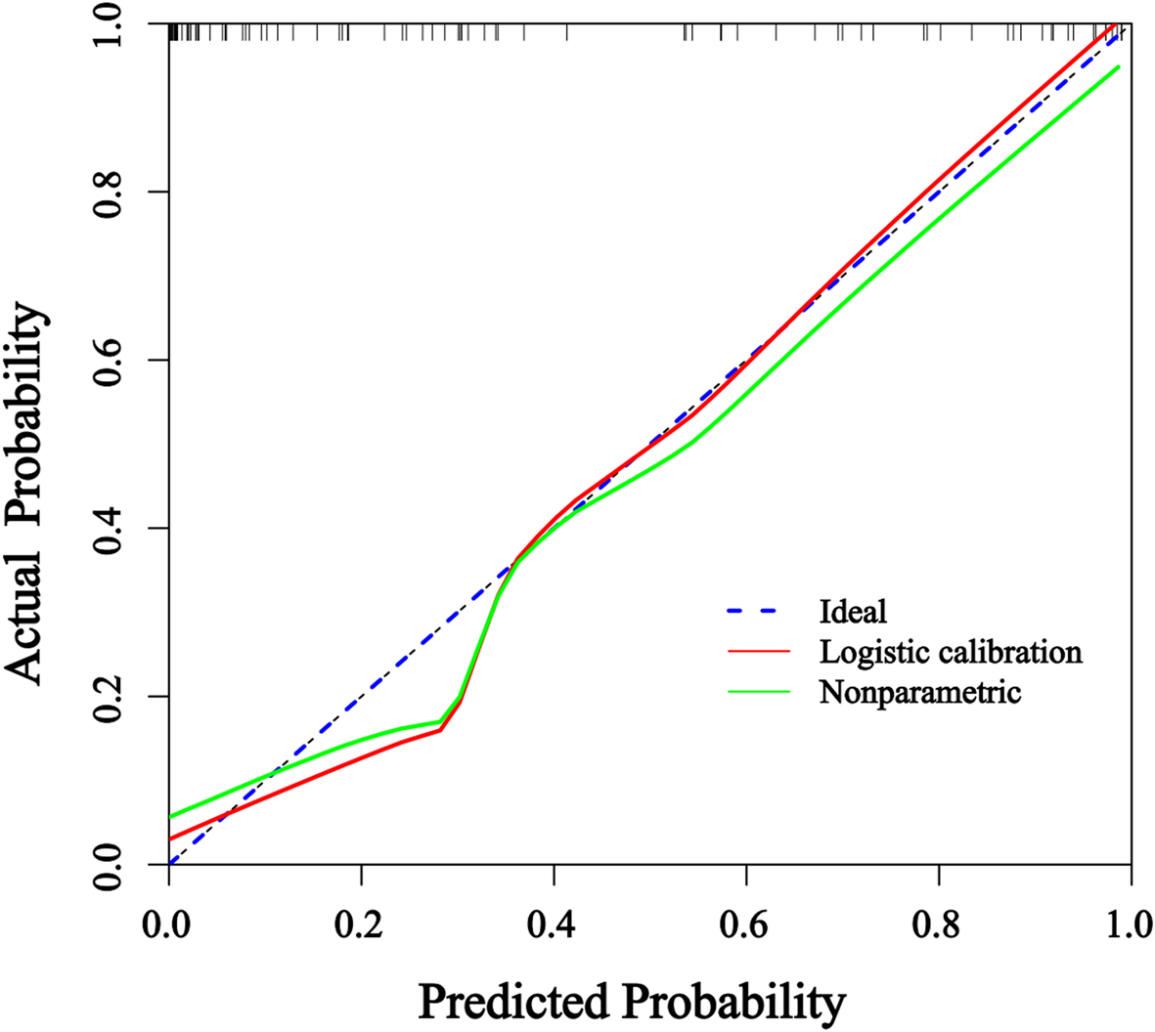
Calibration curves of the nomogram

**Fig. 5:**
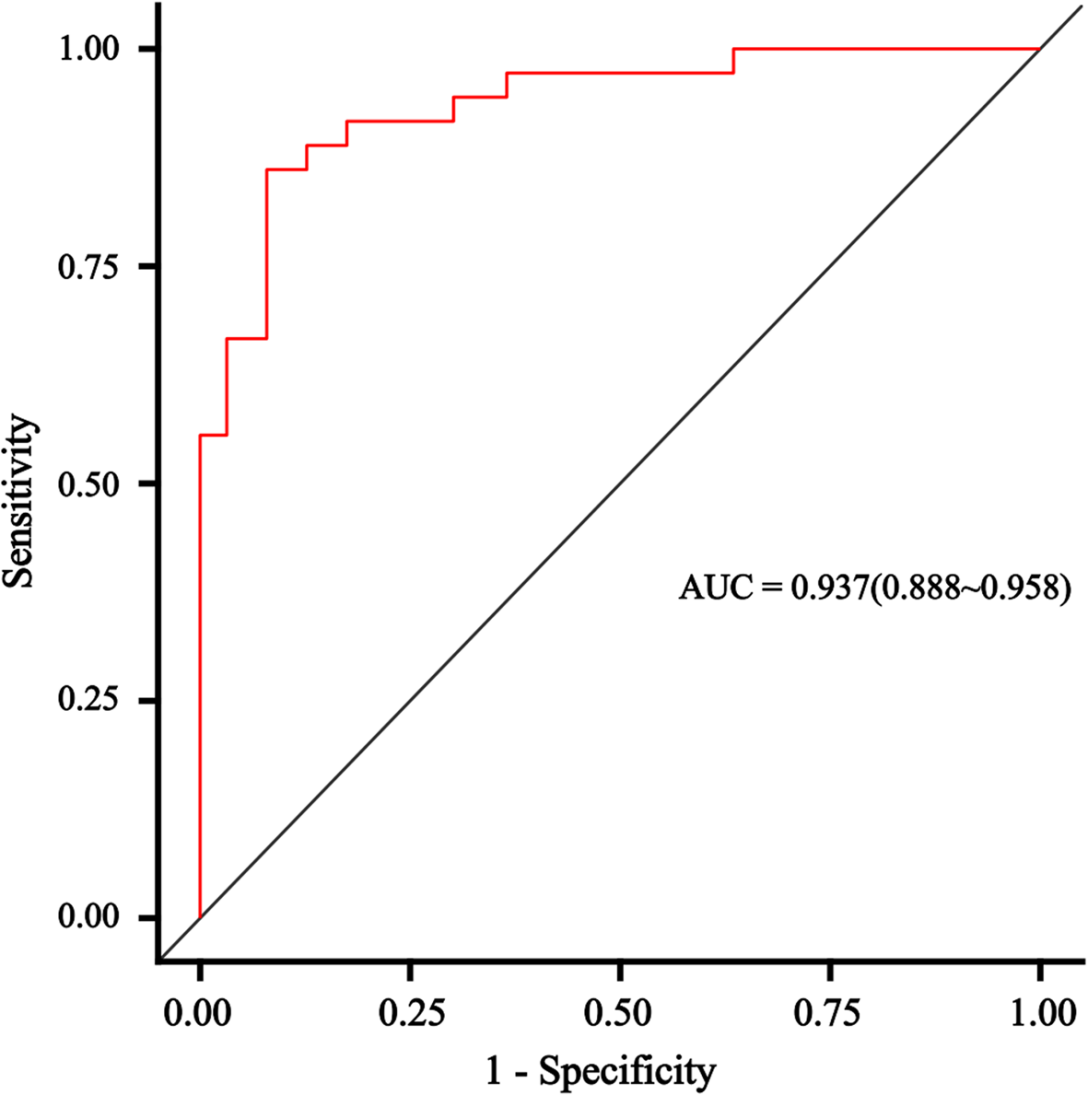
The ROC curves of the nomogram

**Fig. 6:**
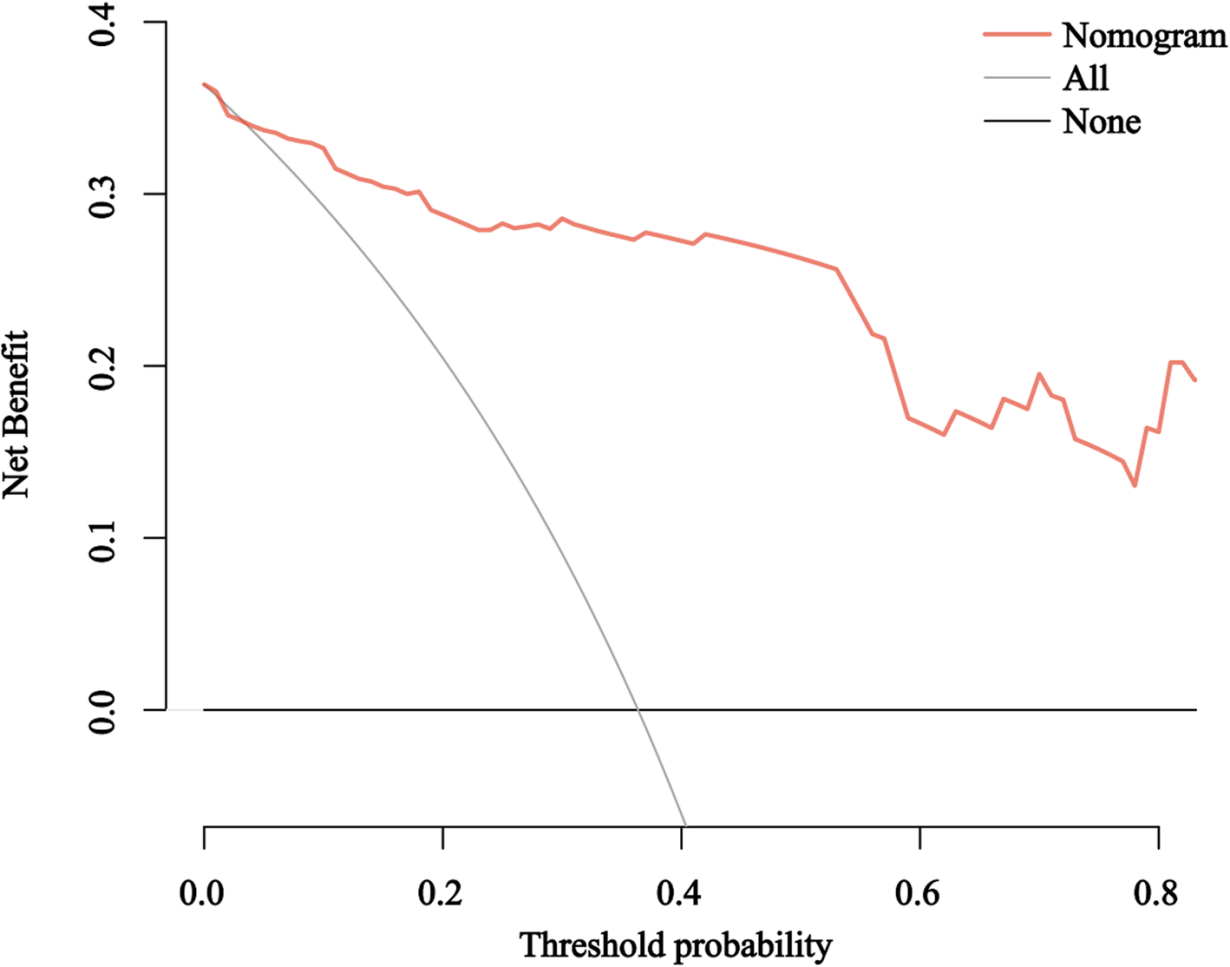
DCA of the nomogram

In order to predict the probability of RIHT individually, the nomogram was constructed on the basis of the results of multivariate logistic regression analysis, including the use of chemotherapy, thyroid volume, Dmean, VS_45_, VS_50_, VS_60_, V_30,60_. The possibility of RIHT could be predicted accurately for each patient with HNC by adding the score of every risk factor and further obtaining the total scores on the point scale (Fig. 3). Figure 4 demonstrated the calibration curve of the nomogram. An AUC of 0.937(95%CI, 0.888–0.958) also was showed outstanding resolving ability of the nomogram (Fig. 5). The decision curve analysis (DCA) of the nomogram is revealed in Fig. 6.

### Additional section

We reviewed data of the antiTPO and antiTg in order to explore the impact on the development of RIHT. There were 13 patients with the data of the levels of antiTPO or antiTg. Among them, 10 patients had normal level of antiTPO and antiTg, and the level of antiTPO and antiTg for other 3 patients is apparently elevated. The incidence of RIHT for patients with abnormal level of antiTPO or antiTg is 66.7%(2/3) and that for those with normal level of relevant antibodies is 40%(4/10). In spite of the small data volume, we could infer that the level of antiTPO or antiTg may be an indispensable factor associated with RIHT.

## Discussion

RIHT is one of the most common complications observed in patients with HNC that underwent radiotherapy. The median follow-up time for this study was 10 months (rang, 3-30 months), and the incidence of RIHT was 36.9%. Similar phenomenon was found in Sommat et al.’s study [10]. Interestingly, 6 patients occurred hyperthyroidism, in which 3 patients gradually convert to be euthyroid, 1 patient eventually reversed to be hypothyroid. We thought the change from hyperthyroidism to hypothyroidism may be attributed to transient release of thyroid hormone caused by serve injury of thyroid parenchymal cells. The other change from hyperthyroidism to euthyroid could be interpreted by increased permeability of cellular membrane that were not changed [11]. These are good explanations for the transient changes of thyroid function observed in our series patients.

### Clinical factors associated with hypothyroidism occurrence

The significantly related clinical factors for RIHT confirmed in our study were female gender, use of chemotherapy, frequency of chemotherapy, N stage, surgery involving thyroid and smaller thyroid volume. In our study, female patients were more prone to RIHT compared with male (61.9% vs. 31.2%; *P* = 0.01). Although the smaller volume of the female thyroid glands may be able to explain the sex difference, gender has no significant impact on thyroid volume(14.7 ± 8.6 and 12.1 ± 7.0, respectively; *P* = 0.059) in our research. Fan et al. [12] and Jain et al. [13] reported consistent conclusion. Therefore, female patients with HNC may benefit from extra measures to protect their thyroid gland from radiation exposure.

Age’s impact on RIHT is not clear. Lee et al. analyzed 149 patients with NPC and showed that age has no significant effect upon RIHT [14]. Several researches also believed that age’s effect upon RIHT is not significant, which was consistent to our study [15, 16]. However, in Diaz et al.’s research, the risk for increasing age were 0.93 [17]. Also, other study indicated contrary results that increasing age was subject to the higher incidence of RIHT, particularly for patients who were over 60 years old [18]. The different age distribution of the study population may cause the above discrepancy.

It’s of great concern whether chemotherapy has an effect on the incidence of RIHT. Luo et al. found that 24.5% of the 155 patients who received chemotherapy developed RIHT; only 1 of the 19 patients without chemotherapy developed RIHT (5.26%) [19]. In the Multivariate analysis, chemotherapy was indeed an independent risk factor for RIHT in patients with NPC, which is consistent with the results of our study (*P* = 0.007). And we found that frequency of chemotherapy raised the risk of developing RIHT (*P* = 0.021; 48.3% vs. 22%).

Whether patients with HNC undergo surgery before radiotherapy has a certain influence on the occurrence of RIHT. Vogelius et al. indicated that the risk of RIHT increases regardless of whether the surgery involves the thyroid, yet the incidence of RIHT was higher after surgery involving the thyroid, which was inconsistent with our study’s results [20]. Alba et al. also found that laryngeal surgery affects the occurrence of RIHT in a significant way [21]. Most of researchers believe that surgery treatment may decrease the blood supply of the thyroid. In our study, the effect of surgery on RIHT is not significant, but surgery involving thyroid significantly affect the development of RIHT (*P* = 0.027; 34.7% vs. 83.3%), which accurately show that the impact of surgery on RIHT is attributed to the direct destruction of thyroid by the surgery.

N stage is usually a vital risk factor for RIHT in most studies. Zhou et al. found that patients with advanced N-staging (N2-N3) nasopharyngeal carcinoma had a 0.91-fold increased risk of RIHT compared with patients with early N-staging (N0-N1) (37.38% vs. 13.11%) [22]. Similar conclusion was found in our study: advanced N-staging (N2-N3) significantly increased incidence of RIHT (*P*= 0.050; 29% vs. 48.6%). Because the distance between metastatic lymph nodes and the thyroid may be more closer for patients with advanced N-stage [23]. We should think highly of the change of thyroid function for patients with advanced N stage after radiotherapy.

Many studies have found a clear association between thyroid volume and RIHT: the incidence of RIHT increases with the decrease in thyroid volume. Diaz et al. reported that the incidence of RIHT decreased by 0.93 times for every 1 cm^3^increase in thyroid volume (95% CI, 0.88–0.98) [17]. In a retrospective research of 206 patients with nasopharyngeal carcinoma undergoing radiotherapy, thyroid volume ≤ 12.82 cm^3^ was an independent risk factor for RIHT: when the thyroid volume was less than or equal to 12.82 cm^3^, the incidence of hypothyroidism was 75%, and when the volume was greater than 12.82 cm^3^, the incidence of hypothyroidism was 37.31% [22]. Similarly, we found that thyroid volume ≤ 10.6cm^3^ was an independent risk factor of development of RIHT(*P* = 0.000; 72.5% vs. 14.8%). However, this study also found no significant correlation between thyroid volume and RIHT when patients with thyroid volume ≤ 10.6 cm^3^ were excluded (*P* = 0.304) (Table 6). Thus thyroid volume may be a confounding factor in the risk factors for RIHT. Chyan et al. suggested that thyroid volume may have an impact on dose limitation [24]. For patients with thyroid volume larger than 8cm^3^, setting the thyroid dose to VS_45_ ≧3cm^3^ could decline the incidence of RIHT. If the volume of thyroid is less than 8cm^3^, more rigorous restriction are required, including Dmean < 49 Gy, V_50_ < 45%, VS_45_ ≧3cm^3^ and VS_50_ ≧3cm^3^.

**Table 6:** Analysis between thyroid volume and hypothyroidism

Variable	Euthyroid	Hypothyroid	*P*
Volume > 10.6 cm^3^	52	9	0.304

*Abbreviations*: *Statistically significant at the level of 5%

### Dosimertric factors associated with hypothyroidism occurrence

This study found that Dmean, VS_45_, VS_50_, VS_60_ and V_30,60_ were independent predictors. Many study stated a dose-dependent risk of RIHT. Many investigators believed that the mean dose of thyroid (Dmean) may be the most potential dosimetric factor. Zhai et al. showed that Dmean has an impact on the occurrence of RIHT for patients with nasopharyngeal carcinoma. Grouping according Dmean, they found that patients with Dmean > 45 Gy are almost five times more likely to experience RIHT than those with Dmean ≤ 45 Gy [25]. Our results were similar to the above study. We showed that Dmean > 47.3 Gy was an independent predictor of RIHT ( 96.3% vs 23.1%). What’s more, the threshold of Dmean for the thyroid was aimed to be lower than 50 Gy in the latest international guideline [26].

Some scholars insisted that the thyroid volume which were not disturbed by radiation is accountable for the generation of thyroid hormone and stands for thyroid hormone reserve[14]. We believe that VS_x_ is better than Vx in predicting development of RIHT. Our study suggested at least 8.4cm^3^ of thyroid should be spared form doses exceeding 60 Gy, at least 2.1cm^3^ be spared form doses exceeding 45 Gy and at least 4.9cm^3^ be spared from doses exceeding 50 Gy to decrease the occurrence of RIHT among HNC patients. After comparing AUC value, it was found that VS_60_ was more important in predicting RIHT, which was coordinated with results of Lee et al.’study [14]. They suggested patients with thyroid VS_60_ > 10cm^3^ had more latency and lower incidence of hypothyroidism. The other study also recommended that VS60 ≥ 10cm^3^could become a viable dose limitation [27]. Chow et al. identified 29 relevant studies involving 4,530 patients with HNC and also showed that VS60 > 10cm^3^could be beneficial to decrease the incidence of RIHT [28].

Although the parameter V_x_ can portray the dose distribution of thyroid well, repeated information exists among different V_x_. Peng et al. believed that a new parameter V_a,b_ could be capable to decrease collinearity among dosimetry parameters in some degree. They analyzed 545 with NPC and suggested that V_30,60_ ≦80% might be a practical dose constraint to conduct during IMRT planning for patients with thyroid volume ≦20cm^3 ^[29]. Yet Our study also showed that V_30,60_ was a reliable predictor of hypothyroidism after radiotherapy, we recommended that the threshold of V_30,60_ of 57% could cut down the occurrence of RIHT. As is shown in Fig. 2, those with V_30,60_ ≦57% had markedly lower incidence of RIHT than those with V_30,60_ > 57%. All these researches offered us various dose limit targets. However, researches on the optimal dose threshold of the thyroid has not reached consensus.

We established the nomogram in order to predict the probability of RIHT individually. What’s more, the combined use of chemotherapy, the thyroid volume, Dmean, VS_40_, VS_50_, VS_60_ and V_30,60_in the nomogram demonstrated prominent predictive ability and calibration. Compared with other nomograms, the nomogram showed better predictive ability, and its AUC of 0.937(95%CI, 0.888–0.958) was apparently larger [30, 31].

However, our research has some limitations. First, the follow-up time is short, only 10 months. Second, with a small amount of data about the antiTPO and antiTg, we found that patients with abnormal level of antiTPO and antiTg were more susceptible to RIHT. Thus we inferred that the level of antiTPO or antiTg may be significant to the development of RIHT and should be included in the nomogram. As is known to all, hashimoto disease is subclinical and underdiagnosed but highly prevalent. One of the reasons of RIHT is that radiation may induce the autoimmune reaction of the thyroid. However, the presence of inflammation of the thyroid may trigger more RIHT on the basis of the thyroid undergoing radaition. A research with a great quantities of the data of relevant thyroid antibodies should be conducted to probe into the correlation. In addition, internal and external data validation are needed to further identify the viability of the nomogram.

## Conclusions

Thyroid volume and thyroid VS_60_ are optimal predictors of RIHT in patients with HNC. Moreover, more attention should be paid to patients with thyroid volume ≤ 10.6cm^3^. Thyroid VS_60_ > 8.4cm^3^ may be a useful threshold for predicting the development of RIHT. The nomogram conducted by the research may become a potential and valuable tool to individually predict the risk of RIHT for HNC patients.

## Data Availability

Research data are stored in an institutional repository and will be shared upon request to the corresponding author.

## Abbreviations

RIHT: Radiation-Induced Hypothyroidism
IMRT: Intensity-Modulated Radiotherapy
HNC: Head and Neck Cancer
Dmean: the mean dose of thyroid
NPC: Nasopharyngeal carcinoma
DVHs: Dose-volume histograms
TSH: Thyroid-stimulating hormone
FT3: Free triiodothyronine
FT4: Free thyroxine
ROC: Receiver operating characteristic
EPID: Electronic Portal Imaging Device
CT: Computed tomography
